# Hydrophilic or Lipophilic Statins?

**DOI:** 10.3389/fcvm.2021.687585

**Published:** 2021-05-20

**Authors:** Elisenda Climent, David Benaiges, Juan Pedro-Botet

**Affiliations:** ^1^Department of Endocrinology and Nutrition, Hospital del Mar, Barcelona, Spain; ^2^Department of Medicine, Universitat Autònoma de Barcelona, Campus Universitari Mar, Barcelona, Spain; ^3^Institut Hospital del Mar d'Investigacions Mèdiques (IMIM), Barcelona, Spain

**Keywords:** adverse effects, cardiovascular disease, hydrophilic, lipophilic, pleiotropic effects, statins

## Abstract

Drugs can be classified as hydrophilic or lipophilic depending on their ability to dissolve in water or in lipid-containing media. The predominantly lipophilic statins (simvastatin, fluvastatin, pitavastatin, lovastatin and atorvastatin) can easily enter cells, whereas hydrophilic statins (rosuvastatin and pravastatin) present greater hepatoselectivity. Although the beneficial role of statins in primary and secondary cardiovascular prevention has been unequivocally confirmed, the possible superiority of one statin or other regarding their solubility profile is still not well-established. In this respect, although some previously published observational studies and clinical trials observed a superiority of lipophilic statins in cardiovascular outcomes, these results could also be explained by a greater low-density lipoprotein cholesterol reduction with this statin type. On the other hand, previous studies reported conflicting results as to the possible superiority of one statin type over the other regarding heart failure outcomes. Furthermore, adverse events with statin therapy may also be related to their solubility profile. Thus, the aim of the present review was to collect clinical evidence on possible differences in cardiovascular outcomes among statins when their solubility profile is considered, and how this may also be related to the occurrence of statin-related adverse effects.

## Hydrophilicity vs. Lipophilicity

The classification of drugs as hydrophilic or lipophilic depends on their ability to dissolve in water or in lipid-containing media. In this respect, absorption is faster in lipophilic drugs, whereas the ease for renal excretion is greater in hydrophilic medications.

As most drugs are weak acids or bases, in an aqueous solution they can be present in two phases: ionised or polar and non-ionised or non-polar. The ionised polar fraction is water-soluble whereas the non-ionised is fat-soluble and is the only one that diffuses easily through cell membranes. The degree of molecule ionisation depends on three main factors: their acidic/basic nature, the dissociation constant of the molecule measured by pK and the pH of the medium where it is found. However, the latter is the definite determinant of the drug's availability to cross cell membranes and act on diverse tissues. Moreover, the degree of digestive absorption and renal excretion of each drug can be modified by changing the pH of the medium where it is found (1).

It must also be considered that hydrophilic substances can be excreted without undergoing any transformation. On the other hand, although the kidney can poorly filter ionised molecules (lipophilic), these are mostly reabsorbed back into the tubule. For this reason, most lipophilic substances are metabolised to become more polar metabolites, which then become water-soluble.

Thus, the hydro- or liposolubility of each drug is an aspect that should be carefully considered in clinical practise when deciding on the type of treatment, together with dose adjustment in patients with renal failure. In that clinical scenario, lipophilic drugs should be avoided, although other clinical characteristics must also be considered.

## Hydrophilic vs. Lipophilic Statins

Since the introduction of lovastatin in 1987 as the first 3-hydroxy-3-methyl-glutaryl-coenzyme A (HMG-CoA) reductase inhibitor approved for human therapy, statins have become the most widely used lipid-lowering drugs with proven effect in cardiovascular disease prevention in different clinical settings (2–5). Statins have been classified in 3 categories based on their potency and efficacy in lowering low-density lipoprotein (LDL) cholesterol concentrations. First-generation statins included lovastatin, pravastatin and fluvastatin, simvastatin and atorvastatin belong to the second generation, and rosuvastatin and pitavastatin to the third.

**Figure 1:**
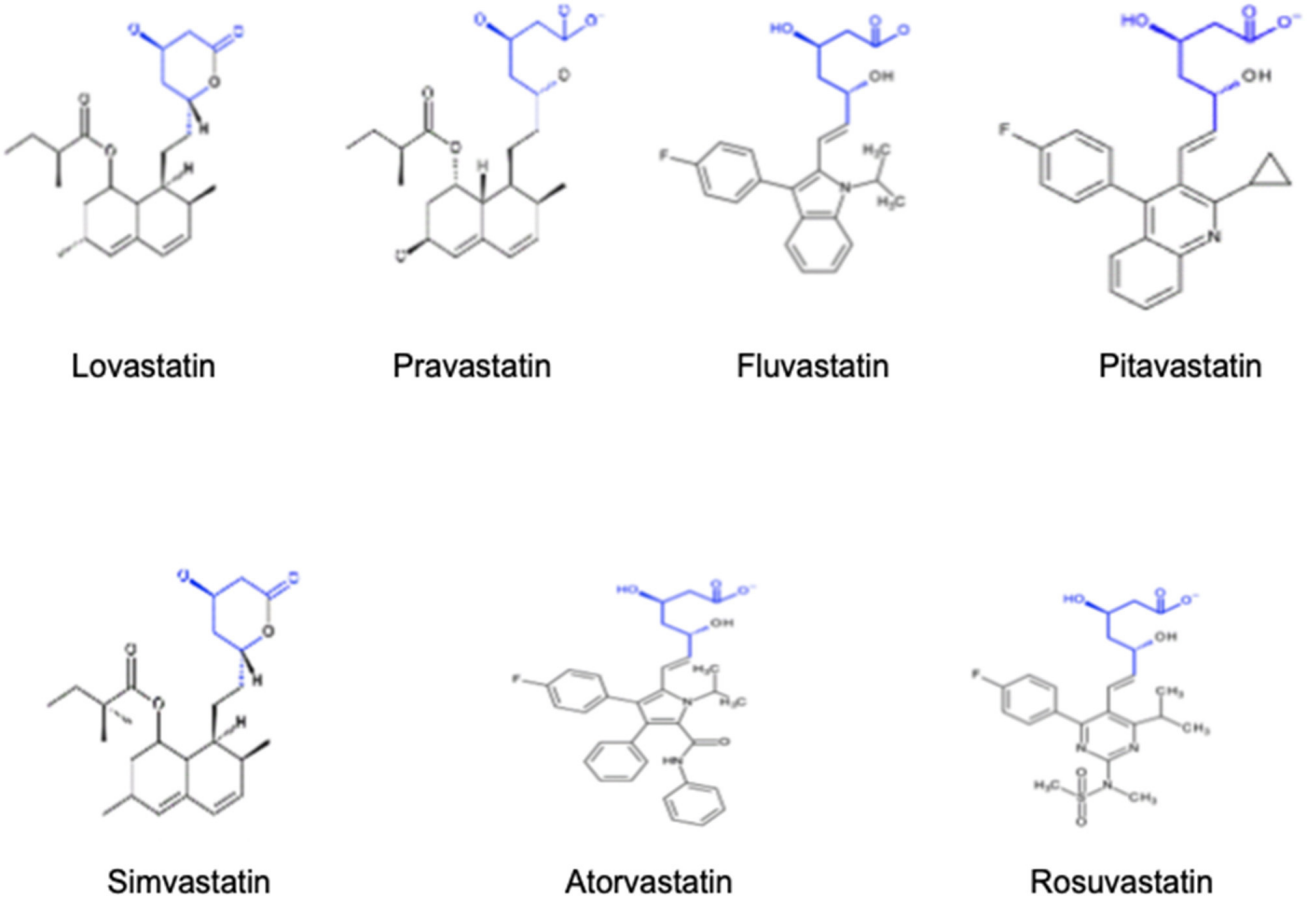
Chemical structure of hydrophilic and lipophilic statins.

Structurally, statins have three main parts as detailed in Figure 1: the analogue of HMG-CoA, a complex ring structure which binds the statin molecule to the HMG-CoA reductase enzyme and, finally, a side chain ring structure that determines structure solubility. They reduce LDL cholesterol levels by inhibiting the rate-limiting enzyme for cholesterol biosynthesis, HMG-CoA reductase. This is a membrane-associated protein located in the endoplasmic reticulum, and the interaction of statins with membranes is likely to influence their ability to inhibit this enzyme. Statins differ markedly in their solubility owing to the presence/absence of polar moieties on the largely hydrophobic backbones. These structural variations result in a differential distribution within the phospholipid bilayer of the cell membranes. According to their solubility, statins can be categorised as hydrophilic and lipophilic. The predominantly lipophilic statins (simvastatin, fluvastatin, lovastatin, pitavastatin, and atorvastatin) can easily pass more deeply into the membranes where they interact with the surrounding acyl chains. By contrast, hydrophilic agents (pravastatin and, to a lesser degree, rosuvastatin) remain associated with the polar surface of the membrane and require protein transporters to enter the cell to inhibit the HMG-CoA reductase enzyme (6). The distinct solubility and location are also likely to be key factors involved in the metabolic differences among statins. Lipophilic statins can enter cells by passive diffusion and therefore become widely distributed in different tissues. However, the liver-specific, carrier-mediated mechanisms required for hydrophilic statin uptake could potentially reduce their ability to exert non-LDL effects at extrahepatic sites. Furthermore, the elimination of statins is also affected by solubility since lipophilic statins are metabolised by membrane-bound cytochrome P450 (CYP) enzymes, whereas hydrophilic statins are mostly eliminated without modification. The main characteristics of each statin type, including dose range, bioavailabilty, liver metabolisation and other relevant aspects are further detailed in Table 1. As an attempt to improve statin bioavailability and effectiveness, a wide variety of polymer- and nanoparticle-based approaches for statin delivery have been described (7, 8).

**Table 1:** Main characteristics of the different statins available in clinical practise.

	**Lovastatin**	**Fluvastatin**	**Pitavastatin**	**Simvastatin**	**Atorvastatin**	**Rosuvastatin**	**Pravastatin**
Dose range (mg/daily)	10–80	20–80	1–4	5–40	10–80	5–40	20–80
Bioavailability (%)	<5	6	>60	<5	12	20	17
Active metabolites	Yes	No	No	Yes	Yes	Yes (minimal)	No
Protein binding (%)	>95	98	96	95	≥90	89	50
Half-life (hours)	2	4.7	12	1–2	14	19	1–2
Faecal excretion (%)	83	90	75	58	90	90	71
Renal excretion (%)	10	<6	2	13	<2	10	20
Liver metabolisation	CYP450 3A4	CYP450 2C9 (minor)	CYP450 2C9	CYP450 3A4	CYP450 3A4	CYP450 2C9 and 2C19	Sulphation
Solubility	Lipophilic	Lipophilic	Lipophilic	Lipophilic	Lipophilic	Hydrophilic	Hydrophilic

Sufficient evidence of the possible beneficial or harmful effects related to statin solubility is lacking. It has been speculated that the ability of lipophilic statins to reach extrahepatic tissues could account for the more favourable cardiovascular outcomes in subjects receiving this type of lipid-lowering drug, although with a higher risk of adverse effects such as statin-associated muscle symptoms (SAMS).

The present comprehensive review aimed to describe the clinical evidence available to date regarding the possible differences in cardiovascular outcomes among statins when their solubility profile is considered, and how this may also be related to statin-related adverse effects. We hope this review will aid better understanding of statins for physicians and ultimately be useful for precise statin selection since, though they share the same mechanism of action, different chemical and pharmacological characteristics may affect their therapeutic efficacy.

## Statin Solubility and Cardiovascular Outcomes

**Figure 2:**
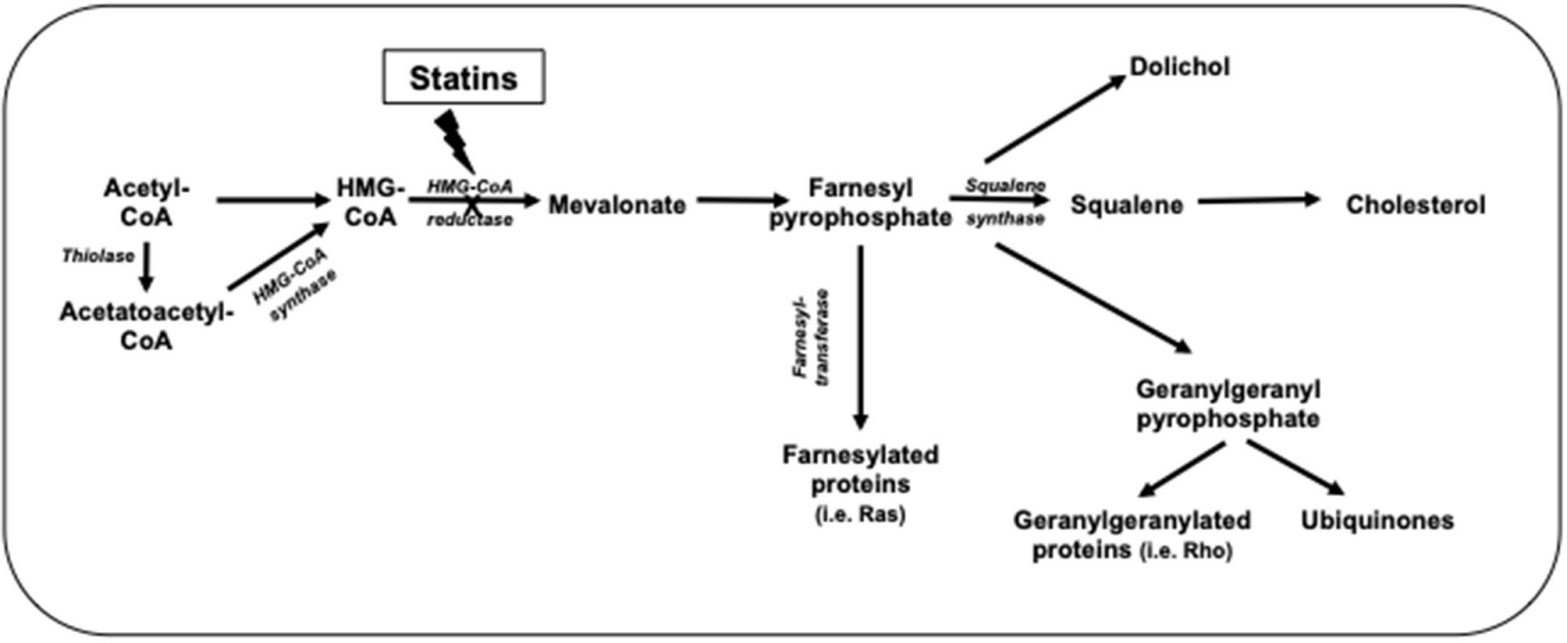
Cholesterol biosynthetic pathway.

**Figure 3:**
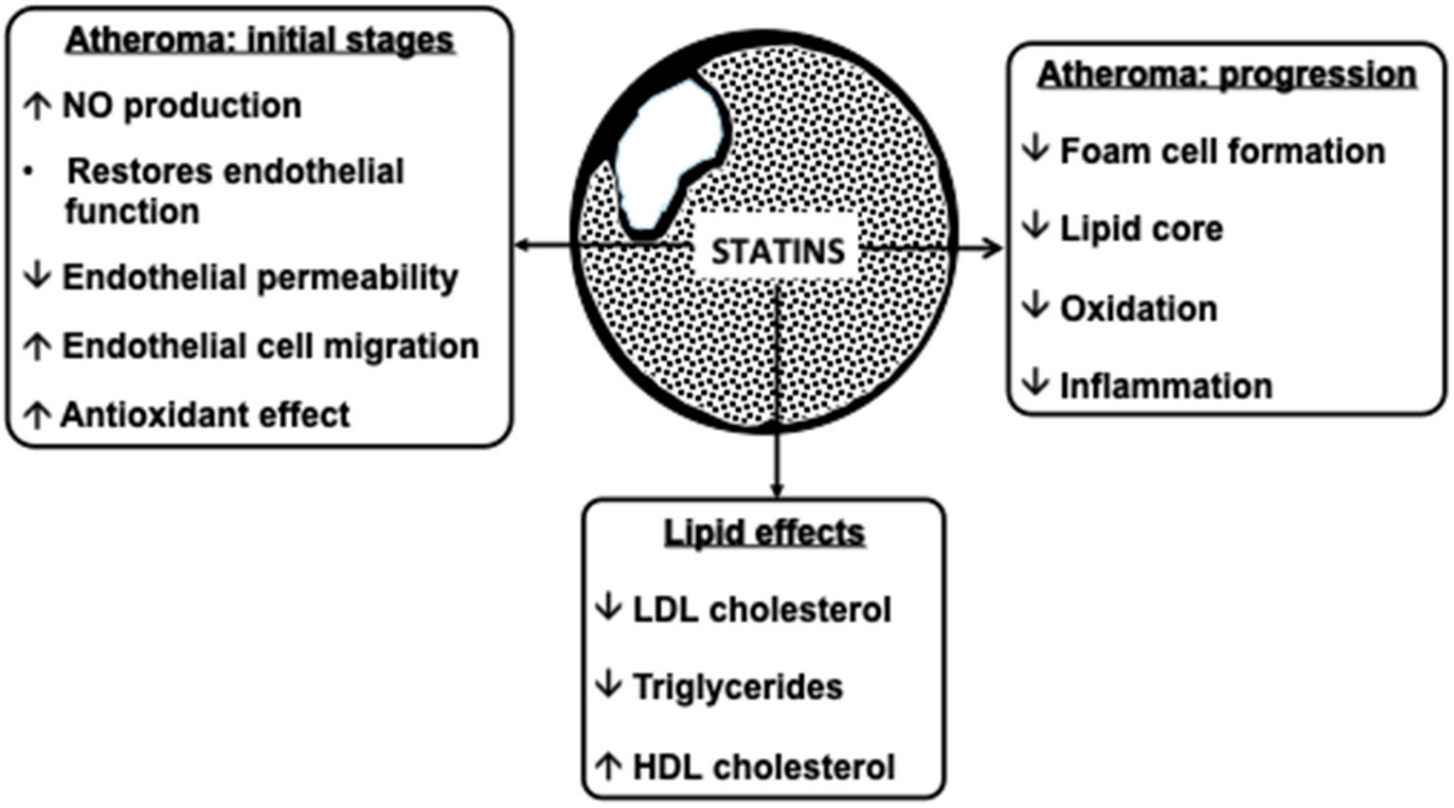
Atheroprotective effects of statins. NO, nitric oxide; LDL, low-density lipoprotein; HDL, high-density lipoprotein.

Statins inhibit cholesterol synthesis, thereby enhancing LDL clearance from the circulation. Since mevalonic acid is the precursor of numerous metabolites, HMG-CoA reductase inhibition potentially results in pleiotropic effects that may affect several tissue functions and modulate specific signal transduction pathways (Figure 2). These effects include anti-inflammatory and antioxidant activities, improvement in endothelial function, increased bioavailability of nitric oxide and delay in the progression of atherosclerotic plaques (9) (Figure 3). All these vasculoprotective effects may account for a greater magnitude of and earlier time to cardiovascular benefit than apparently explained by changes in LDL cholesterol levels alone. However, leaving aside *in vitro* and experimental studies, it is not possible from available clinical evidence to isolate the potential benefits of pleiotropic effects of statins from those conferred by LDL cholesterol reduction.

One of the factors to be considered when evaluating outcomes of lipid-lowering treatment with statins is their solubility. Therefore, the role of both hydrophilic and lipophilic statins regarding beneficial pleiotropic effects and thus a possible improvement in cardiovascular primary or secondary prevention needs to be analysed further.

### Statins and Heart Failure

According to the 2016 European Society of Cardiology definition (10), heart failure (HF) is a complex disease caused by structural and/or functional cardiac abnormality, resulting in reduced cardiac output and/or elevated intracardiac pressures at rest or during stress. Its management is challenging owing to the clinical heterogeneity of the disease, which leads to patients responding differently to evidence-based standard therapy. Furthermore, its prevalence has suffered an exponential increase in the last decade, rendering HF a serious public health issue (11).

The role of statin therapy in HF remains controversial, with conflicting findings from observational studies and clinical trials (12, 13). Next, we will analyse its impact in two different clinical settings: the prevention of HF and the treatment of established HF.

#### Statins and Incident HF

Some trials (including both hydrophilic and lipohilic statins) on cardiovascular prevention reported interesting findings in the HF field. In this respect, those which compared statin vs. placebo (14, 15) or more-intensive vs. less-intensive statin therapy (16–19) found an HF incidence reduction in patients with stable coronary heart disease or a history of acute coronary syndrome without previous HF. Thus, efficacy in myocardial ischaemic event reduction could be involved in their benefit in incident HF prevention.

A large-scale meta-analysis of randomised primary and secondary cardiovascular prevention clinical trials with statins showed a modest reduction (10%) in first non-fatal HF hospitalisation with statin therapy, with no effect on HF death. However, no differences were found in risk reduction between patients who presented an incident myocardial infarction or not. Only 10–15% of non-fatal HF hospitalisations were preceded by a documented within-trial non-fatal myocardial infarction (20). Although the mechanisms by which statins reduce non-fatal HF hospitalisations are not well-established, the lack of a significant effect on HF mortality may be attributed to a non-ischaemic cause of death in HF patients.

More recently, Imran et al. (21), evaluating nearly 8 million subjects in an observational cohort study, reported a reduction in HF in those treated with hydrophilic compared to lipophilic statins which seemed to be driven by high-dose rather than low-dose hydrophilic statins. This was the only large cohort study using health care data designed to compare the risk of incident HF between hydrophilic and lipophilic statins.

In summary, more evidence is needed to support the use of high-intensity hydrophilic statins in the context of incident HF prevention.

#### Statins in the Treatment of Established HF

Data from the major lipid-lowering trials on the effects of statin therapy on prevalent HF are scant since the majority excluded patients with this syndrome (22, 23).

Beside small studies with atorvastatin (lipophilic) (24–26) or other statins (27) suggesting a potential benefit of these therapies in HF patients, two randomised controlled trials with rosuvastatin (hydrophilic), GISSI (Gruppo Italiano per lo Studio della Sopravvivenza nell'Insufficienza cardiaca) (28) and CORONA (Controlled Rosuvastatin Multinational Trial in Heart Failure) (17) were specifically conducted in an HF population. No differences in major cardiac adverse events were registered when compared to placebo; however, a reduction in HF hospitalisation was observed in CORONA. Furthermore, in a pooled analysis of both trials, Feinstein et al. (29) found a small but significant risk reduction in myocardial infarction in ischaemic HF patients treated with the hydrophilic rosuvastatin.

Data on comparative effects between lipophilic and hydrophilic statin exposure for HF-related outcomes are limited. Using an indirect comparison approach, the meta-analysis of Bonsu et al. (30), including 13 studies and 10,966 patients, reported a superiority of lipophilic statins regarding all-cause mortality [odds ratio (OR) 0.50; 95% confidence interval (CI): 0.11–0.89; *p* = 0.01], cardiovascular mortality (OR: 0.61; 95% CI: 0.25–0.97; *p* = 0.009) and hospitalisation for worsening HF (OR 0.52; 95% CI, 0.21–0.83; *p* = 0.0005); however, both statin types were comparable for cardiovascular hospitalisation (OR: 0.80; 95% CI: 0.31–1.28; *p* = 0.36). Similarly, other studies also showed a greater risk reduction in HF events, hospitalisation and mortality (cardiovascular and all-cause) with lipophilic vs. hydrophilic statins (20, 31–34). It has been postulated that the greater exposure of lipophilic statins in extrahepatic tissues could account for a higher uptake by cardiac muscle (35).

The supposed superiority of lipophilic statins has also been observed when cardiac function and anti-inflammatory effects were evaluated in patients with established HF. In this respect, Bonsu et al. (36) in another meta-analysis with 19 randomised controlled trials and ~6,200 patients obtained more favourable results with lipophilic statins in improving cardiac function and reducing inflammation, with a greater rise in left ventricular ejection fraction. Lipophilic statins were also superior to hydrophilic rosuvastatin and pravastatin regarding B-type natriuretic peptide (BNP), high sensitivity C-reactive protein (hsCRP), interleukin 6 and tumour necrosis factor α reductions during follow-up. Takagi et al. (37) found lipophilic atorvastatin to be superior to hydrophilic rosuvastatin in improving cardiac function, with superiority of the former in inflammation attenuation and endothelial dysfunction, refuting previous reports, which supported the superiority of rosuvastatin in hsCRP level reductions.

Mortality in patients with HF has also been associated with cardiac sympathetic nerve activity, which in turn is one of the most important prognostic factors (38, 39). In this regard, previous evidence once again showed the superiority of lipophilic atorvastatin vs. hydrophilic rosuvastatin in sympathetic nerve activity reduction in patients with HF (40). In that face-to face trial (40), 5 mg of lipophilic atorvastatin was compared to 2.5 mg of hydrophilic rosuvastatin in patients with dilated cardiomyopathy. As to lipid profile outcome, total and LDL cholesterol reductions were similar between groups. However, the atorvastatin group presented an increased heart/mediastinum ratio (which is known marker of an increased noradrenaline uptake), improved left ventricular ejection fraction, and greater reduction in BNP levels. Hence, beyond improving lipid profile, these findings emphasise the role of statins on inflammation response (41). Moreover, preclinical evidence suggests that the pleiotropic effects of statins may improve survival rates in ischaemic and non-ischaemic HF subjects by regulating the autonomic nervous system through angiotensin II and nitric oxide modulation (42). However, preliminary human studies reported mixed results. Indeed, Horwich al. (43) found that short-term statin treatment failed to result in a significant reduction in autonomic nervous system activation in HF patients. In contrast, a large meta-analysis of 13 randomised trials reported that lipophilic statins significantly lowered all-cause mortality, hospitalisation for worsening HF and LDL cholesterol, regardless of age, baseline left ventricular ejection fraction and cause of HF (32).

Other studies evaluated possible explanations for more favourable results with hydrophilic statins. In this respect, some studies reported that fat-soluble statins induced a pro-apoptotic state in human adult cardiac myocytes *in vitro* (44). Similarly, it has been speculated that lipophilic statins might inhibit CoQ10 biosynthesis, leading to disturbances in cardiac energy metabolism (45). A previous study showed that switching from lipophilic simvastatin to the hydrophilic pravastatin led to an increase in plasma adiponectinaemia with no change in LDL cholesterol concentrations (46).

A recent study comparing the effects of atorvastatin vs. rosuvastatin on left ventricular function, inflammatory and fibrosis biomarkers in patients with chronic HF, published by El Said et al. (47) suggested that the impact of lipophilic atorvastatin was greater than that of hydrophilic rosuvastatin in HF patients with regards to the improvement in left ventricular ejection fraction and soluble suppression of tumorigenicity reduction, a novel fibrosis marker.

Finally, we believe the results of a *post-hoc* analysis of the CORONA trial are worth mentioning (48). Plasma galectin 3 (a mediator of fibrogenesis) levels were evaluated in subjects with established systolic HF included in the CORONA trial that were randomised to 10 mg or rosuvastatin daily or placebo. It was observed that among patients with below the median plasma concentrations of galectin 3 (≤19 ng/ml), those receiving rousvastatin treatment presented a lower primary event rate (defined as cardiovascular death, myocardial infarction or stroke), lower mortality rate and lower rate of all-cause mortality or HF hospitalisation. However, these benefits were not observed in patients with higher galectin 3 levels. Thus, these results may highlight the hypothetical role of galectin 3 to identify subjects with HF or chronic left ventricular systolic dysfuncion that might benefit of statins treatment, although further studies are required.

Thus, to date, the rationale for proving the superiority of one statin type over the other remains unclear. Although some studies have described possible pathophysiological mechanisms that could favour hydrophilic or lipophilic statins regarding HF outcomes, these have not been further confirmed. Future trials with larger sample size and longer follow-up are essential to ascertain whether real differences exist among statins owing to their solubility profile, and hence play a role when the optimal lipid-lowering therapy is decided upon for each patient in clinical practise. Meanwhile, statin use as HF therapy is not recommended.

### Statins and Coronary Heart Disease

As in HF outcomes, previous studies have also yielded conflicting results concerning the possible benefits of different statin types owing to their solubility in primary of CHD prevention and in established cardiovascular disease (49, 50).

#### Primary CHD Prevention

The valuable role of statins in primary CHD prevention has been unequivocally confirmed in previous reports. Data from the Cholesterol Treatment Trialists' (CTT) Collaborators of statin treatment in people at low cardiovascular risk demonstrated a 9% reduction [relative risk (RR): 0.91, 95% CI: 0.85–0.97] in all-cause mortality and 25% (RR: 0.75, 95% CI: 0.70–0.80) in major vascular events per 1.0 mmol/L reduction in LDL cholesterol, even among low-risk patients (4). Furthermore, a 2013 Cochrane analysis corroborated these findings with a 14% reduction (OR: 0.86, 95% CI: 0.79–0.94) in all-cause mortality and 25% (RR: 0.75, 95% CI: 0.70–0.81) in cardiovascular events (51).

Whether the solubility profile of each statin type could account for this favourable cardiovascular benefit remains open to debate. In this respect, the previously mentioned CTT meta-analysis (4) included some studies that compared various statin types; however, the differences observed in cardiovascular outcomes were attributed to statin potency (intensive vs. less intensive), and no mention was made of the solubility profile resulting in more favourable outcomes.

#### Secondary CHD Prevention

The possible differences between hydrophilic and lipophilic statins have mainly been evaluated regarding secondary cardiovascular prevention in patients with acute coronary syndrome and stable CHD (Table 2) (52–59).

**Table 2:** Trials comparing hydrophilic and lipophilic statins and coronary artery disease.

**Study (reference)**	**Study design**	**Follow-up (months)**	**Trial comparison**	**Primary endpoints**	**Main results**
PROVE IT-TIMI 22 (52)	RCT, double-blind	24	Pravastatin 40 mg vs. atorvastatin 80 mg	MACE	MACE 26.3% after pravastatin and 22.4% after atorvastatin; *p* = 0.005
REVERSAL (53)	RCT, double-blind	18	Atorvastatin 80 mg vs. pravastatin 40 mg	Percentage change in total atheroma volume	Significantly lower progression rate of atheroma volume in atorvastatin group (*p* = 0.02). Coronary atherosclerosis progression in pravastatin group (2.7%; 95% CI, 0.2–4.7%; *p* = 0.001) compared with baseline. No progression in atorvastatin arm (−0.4%; CI −2.4 to 1.5%; *p* = 0.98) compared with baseline.
SAGE (54)	RCT, double-blind	12	Atorvastatin 80 mg vs. pravastatin 40 mg	Total duration of ischaemia on 48 h holter- monitor	Absolute change from baseline in total duration of ischaemia at month 12 significantly reduced in both groups (*p* < 0.001 for each treatment group) with no significant difference between treatment groups. Atorvastatin greater LDL cholesterol reductions than pravastatin, trend towards fewer MACE (hazard ratio, 0.71; 95% CI, 0.46–1.09; *p* = 0.114), and a significantly greater reduction in all-cause death (hazard ratio, 0.33; 95% CI, 0.13–0.83; *p* = 0.014).
MUSASHI-AMI (55)	RCT, double-blind	24	Lipophilic (atorvastatin, simvastatin, pitavastatin, fluvastatin) vs. hydrophilic (pravastatin)	CV death, non-fatal MI, recurrent acute myocardial ischaemia	Although LDL cholesterol was reduced more potently in the lipophilic group (−34 vs. −19%; *p* = 0.0069), ACS tended to occur less frequently (3.6 vs. 9.9%; *p* = 0.0530) and the incidence of new Q-wave in ECG was significantly lower (75 vs. 89%; *p* = 0.0056) in the hydrophilic group.
CENTAURUS (56)	RCT, double-blind parallel group trial	3	Atorvastatin 80 mg vs. rosuvastatin 20 mg	Percentage change in ApoB/ApoA-1 ratio	Rosuvastatin 20 mg was more effective than atorvastatin 80 mg in decreasing apoB/apoA-1 ratio at 1 month (−44.4 vs. −42.9%, *p* = 0.02), but not at 3 months (both −44.4%, *p* = 0.87).
LUNAR (57)	RCT, open-label, parallel group trial	3	Atorvastatin 80 mg vs. rosuvastatin 20–40 mg	Change in LDL cholesterol	Rosuvastatin 40 mg efficacy in lowering LDL cholesterol levels was significantly greater vs. atorvastatin 80 mg (46.8 vs. 42.7% decrease, *p* = 0.02). Comparable results for rosuvastatin 20 and atorvastatin 80 mg. Increase in HDL cholesterol was significantly greater with rosuvastatin 40 (11.9%, *p* < 0.001) and 20 mg (9.7%, *p* < 0.01) than with atorvastatin 80 mg (5.6%).
The ROMA II (58)	RCT, double-blind	12	Atorvastatin 80 mg vs. rosuvastatin 40 mg vs. controls on chronic statin therapy without reloading	Incidence of peri- procedural MI, MACE	12 and 24-h post-PCI CK-MB elevation >3 × occurred more frequently in control than in the rosuvastatin and atorvastatin groups (at 24-h: 25.0 vs. 7.1; *p* = 0.003 and 25.0 vs. 6.1; *p* = 0.001). At 30-day, 6- and 12-month follow-up, the incidence of MACE was higher in the control group than in the rosuvastatin or atorvastatin groups (at 12-month: 41.0 vs. 11.4 vs. 12.0%; *p* = 0.001).
ALPS-AMI (59)	RCT, open -label, blinded-endpoint	24	Atorvastatin 10–20 mg vs. pravastatin 10–20 mg	All-cause death, CV death, MI, stroke, revascularisation, hospitalisation	Primary endpoint occurred in 77 (30.4%) and in 80 patients (31.4%) in the pravastatin and atorvastatin groups, respectively (hazard ratio, 1.181; 95% CI: 0.862–1.619; *p* = 0.299), whereas greater reductions in total and LDL cholesterol were achieved in the atorvastatin group (*p* < 0.001 for each).

*ACS, acute coronary syndrome; CI, confidence interval; CV, cardiovascular; ECG, electrocardiogram; MACE, major adverse cardiovascular event; MI, myocardial infarction; PCI, percutaneous coronary intervention; RCT, randomised controlled trial*.

Back in 2004, the PROVE IT-TIMI 22 trial (52) compared a standard therapy of 40 mg pravastatin daily with an intensive therapy of 80 mg atorvastatin daily, observing that an intensive lipid-lowering statin strategy provided greater protection against death or major adverse cardiovascular events (MACE) than a standard regimen. Similarly to the results from observational studies, the greater LDL cholesterol reduction with lipophilic atorvastatin compared to hydrophilic pravastatin could probably account for the more favourable cardiovascular results observed in subjects receiving the former, although whether the solubility profile of each statin could also play a role in these observed differences could also be speculated.

Kim et al. (60) found a higher composite of MACE in the hydrophilic statin group at 1 and 6 months (1 month: 10.0 vs. 4.4%; *p* = 0.001; 6 months: 19.9 vs. 14.2%; *p* = 0.022), whereas no significant difference in MACE was observed at 1 year of follow-up (21.5 vs. 17.9%; *p* = 0.172). Both statin arms showed similar efficacy in the lipid profile and the use of a hydrophilic statin did not predict 1-year MACE, all-cause death, acute myocardial infarction or re-percutaneous coronary intervention.

To shed light on this matter, further trials such as that reported by Sakamoto et al. (55) in 2007 directly compared the lipophilics atorvastatin, fluvastatin, pitavastatin and simvastatin to hydrophilic pravastatin. Although LDL cholesterol was reduced more potently in the lipophilic than the hydrophilic groups (−34 vs. −19%; *p* = 0.0069), acute coronary syndromes tended to occur less frequently (3.6 vs. 9.9%; *p* = 0.0530) and the incidence of new a Q-wave appearance on electrocardiogram was significantly lower (75 vs. 89%; *p* = 0.0056) with hydrophilic pravastatin than with lipophilic statins. The CENTAURUS trial (56) also obtained more favourable results with hydrophilic statins, with rosuvastatin 20 mg being more effective than lipophilic atorvastatin 80 mg in reducing the apoB/apoA-1 ratio at 1 month (−44.4 vs. −42.9%, *p* = 0.02), although these results were not further confirmed with a longer follow-up. Finally, Izawa et al. (59) obtained more favourable results with hydrophilic pravastatin (MACE 30.4% after pravastatin vs. 31.4% in the atorvastatin group), although greater reductions in total and LDL cholesterol were achieved in the atorvastatin group (*p* < 0.001 for each).

On the other hand, the findings of Bytyçi et al. (61) between hydrophilic and lipophilic statins were comparable in terms of risk reduction for MACE (RR: 0.969; 95% CI: 0.835–1.125; *p* = 0.682), myocardial ischaemia (RR: 0.880; 95% CI: 0.731–1.058; *p* = 0.174), cardiovascular death (RR: 0.757; 95% CI: 0.486–1.180; *p* = 0.219) and all-cause mortality (RR: 0.797; 95% CI: 0.590–1.075; *p* = 0.137). However, the cardiovascular hospitalisation rate was lower (RR: 0.789; 95% CI: 0.643–0.969; *p* = 0.024) and alanine aminotransferase (ALT) elevation higher (RR: 2.689; 95% CI: 1.841–3.954; *p* < 0.001) in lipophilic- than in hydrophilic-treated patients.

With regard to coronary atherosclerosis progression/regression studies, the REVERSAL trial (53) assessed the effect of different statin regimens designed to produce intensive or moderate lipid lowering of the coronary artery atheroma burden and progression. Six hundred and fifty-four patients were randomly assigned to receive a moderate lipid-lowering regimen consisting of 40 mg pravastatin or an intensive lipid-lowering regimen with 80 mg atorvastatin. They observed that the percentage change in atheroma volume revealed a significantly lower progression rate in the lipophilic atorvastatin group compared with the hydrophilic pravastatin group (*p* = 0.02). However, as in the CTT meta-analysis (4), this could probably be explained by the fact that the LDL cholesterol reduction was greater in the atorvastatin than the pravastatin groups (*p* < 0.001), and hence the solubility profile of each statin and the possible superiority of lipophilic statins could play a secondary role in the observed differences.

Moving forward, it must be acknowledged that the previously mentioned studies focused mainly on acute coronary syndrome. Here, the possible beneficial effects of one statin type or the other (hydrophilic vs. lipophilic) may probably depend more on their pleiotropic effects since the impact of lipid-lowering therapy in LDL cholesterol reduction has not yet been attained. However, when the focus is on chronic ischaemic heart disease, the beneficial effects of LDL cholesterol reduction may be present to the same degree as the pleiotropic changes. In this respect, Deedwania et al. (54) evaluated 893 outpatients with chronic stable ischaemic heart disease randomised to atorvastatin 80 mg (lipophilic) or pravastatin 40 mg (hydrophilic) and followed for 12 months. The primary efficacy parameter (absolute change from baseline in total ischaemia duration at month 12) was significantly reduced in both groups at months 3 and 12 (*p* < 0.001 for each treatment group) with no significant difference between treatment groups. However, atorvastatin-treated patients had greater LDL cholesterol reductions than pravastatin-treated patients, a trend towards fewer MACE (hazard ratio: 0.71; 95% CI: 0.46, 1.09; *p* = 0.114) and a significantly greater reduction in all-cause death (hazard ratio: 0.33; 95% CI: 0.13, 0.83; *p* = 0.014). Hence, as previously mentioned, the probable superiority of lipophilic atorvastatin could once again be explained by its greater potency in lowering LDL cholesterol concentrations.

Finally, the possible pleiotropic effects that may account for all these observed results include decreased adenosine triphosphate (ATP) production with lipophilic statins and enhanced myocardial stunning after ischaemia and reperfusion (62), with direct beneficial effects on cardiovascular outcomes. Moreover, it has been further observed that lipophilic simvastatin enhances myocardial stunning compared with controls and hydrophilic pravastatin (45). However, both types of statins, apart from their lipid-lowering effect, increase nitric oxide production and release (63), thus protecting the myocardium against ischaemia-reperfusion injury, and reduce infarct size (64, 65).

Nevertheless, while some studies, particularly randomised controlled trials, detected superiority of hydrophilic statins regarding to secondary CHD prevention, others reported greater LDL cholesterol reductions with lipophilic statins, which could also account for the more favourable cardiovascular outcomes. As for HF outcomes, we believe future randomised trials with longer follow-up are mandatory to confirm the possible superiority of one statin type over the other taking into account their solubility profile, and regardless of their intensity in lowering LDL cholesterol levels.

### Statins and Atrial Fibrillation-Related Stroke

The possible differences between statin types and the risk of atrial fibrillation related stroke has also been evaluated. In this sense, a meta-analysis (66) including a total of 8 studies evaluated the clinical outcomes both for pre- and post-stroke statins. They observed that post-stroke statin therapy reduced total mortality regardless of statin intensity. However, no differences were observed regarding statin treatment and a reduction in the risk of recurrent ischaemic stroke. As to pre-stroke statins, initiating lipid-lowering treatment before the event was associated with a lower risk of poor short-tem functional outcomes. Another recent meta-analysis in atrial fibrillation patients conformed a reduction in all-cause and cardiovascular mortality rates (67). Despite these favourable results, possible differences between statins about their solubility profile were not assessed; hence future studies are needed in this field to reach more solid conclusions that can be useful in clinical practise.

## Statin Solubility and Adverse Effects

Although the different statin types have possible beneficial effects depending on their solubility profile, safety cannot be ignored. It has been argued that the benefits of lipophilic statins may transcend into diverse adverse reactions owing to their easy penetration into extrahepatic tissues. However, solid evidence is still lacking in this field (17, 28, 68). On the other hand, the hepatoselectivity of hydrophilic statins could also translate into specific organ damage, although their lower tissue absorption and lower dependence on the cytochrome P450 enzyme compared to lipophilic statins could explain a drop in the number of side effects in subjects treated with these types of statin (69).

Firstly, the prevalence of SAMS differs between statin classes. Lipophilic statins such as simvastatin, atorvastatin, fluvastatin, pitavastatin and lovastatin, owing to their well-known ability to non-selectively diffuse into extrahepatic tissues, such as skeletal muscle, carry a higher risk of SAMS. In contrast, hydrophilic statins have less muscle penetration and therefore lower risk (70). However, based on a recent observational cohort study, equipotent hydrophilic statins were not better tolerated compared to lipophilic statins (71). It must also be considered that, beside the statin type, the risk of SAMS also depends on other factors such as the concomitant use of drugs metabolised by the same hepatic cytochrome P450 isoforms (72). Other risk factors for SAMS to be consideret include family history of muscular symptoms with lipid-lowering therapy, untreated hypothyroidism, female sex, older age and low body mass index, among others (73).

Secondly, the presence of new-onset diabetes mellitus with statin therapy should also be mentioned. This seems to be more frequent in patients with pre-existing risk factors, including metabolic syndrome traits (74). It has been observed for both hydrophilic and lipophilic statins and appears to occur more frequently in older patients and those on high-dose statin therapy; however, a relationship with statin solubility has not been described (70).

As for neurological disorders, it has been hypothesised that lipophilic statins could induce a higher risk given their increased ability to cross the blood-brain barrier (75); however, these findings have not been confirmed in further studies. Furthermore, it should be noted that these effects may not be specific to statin type *per se* but rather result from changes in cholesterol concentrations.

Finally, controversy persists regarding the effects of statins on renal function. Except for hydrophilic pravastatin and rosuvastatin, the remaining statins are mainly metabolised by the liver and minimally cleared by the kidney. Mild transient proteinuria has previously been observed in some patients when receiving high-dose statin treatment; however, this has not been firmly associated with impaired renal function (76).

## Conclusions

The classification of drugs as hydrophilic or lipophilic depends on their ability to dissolve in lipid media or in water. The predominantly lipophilic statins can easily enter cells and interact with cell membranes, whereas hydrophilic statins present greater hepatoselectivity.

Conflicting results have been observed on the superiority of hydrophilic or lipophilic statins regarding cardiovascular outcomes, including HF and CHD, both from primary and secondary prevention. In this respect, the possible superiority of lipophilic statins seen in some studies could be explained by a greater LDL cholesterol reduction with this statin type, with the solubility profile playing a secondary role in the favourable cardiovascular outcomes observed.

Finally, the non-selective diffusion of lipophilic statins into extrahepatic tissues could account for an increase in SAMS, albeit without differences between hydrophilic and lipophilic statins with respect to other adverse effects. Thus, we believe future studies are essential in this field for the solubility profile of statins to be taken into account when deciding on the optimal lipid-lowering therapy for each patient in daily clinical practise.

## Author Contributions

The first version of the manuscript was written by EC, and revised and approved by DB and JP-B. All authors contributed to the article and approved the submitted version.

## Conflict of Interest

The authors declare that the research was conducted in the absence of any commercial or financial relationships that could be construed as a potential conflict of interest.
